# The laboratory findings and different COVID-19 severities: a systematic review and meta-analysis

**DOI:** 10.1186/s12941-021-00420-3

**Published:** 2021-03-16

**Authors:** Erfan Kazemi, Reihane Soldoozi Nejat, Fatemeh Ashkan, Hossein Sheibani

**Affiliations:** 1 Student Research Committee, School of Medicine, Shahroud University of Medical Sciences, Shahroud, Iran; 2 Clinical Research Development Unit, Imam Hossein Hospital, Shahroud University of Medical Sciences, Shahroud, Iran

**Keywords:** COVID-19, Creatinine, Leukocyte, Lymphocyte, Hemoglobin, Platelet, C-reactive protein

## Abstract

**Background:**

Abnormal laboratory findings are common in patients infected with severe acute respiratory syndrome coronavirus 2 (SARS-CoV-2). The aim of this systematic review was to investigate the effect of the level of some laboratory factors (C-reactive protein (CRP), creatinine, leukocyte count, hemoglobin, and platelet count) on the severity and outcome of coronavirus disease 2019 (COVID-19).

**Methods:**

We searched PubMed, Web of Science, Scopus, and Google Scholar. We collected the articles published before May 26, 2020. We gathered the laboratory factors in groups of patients with COVID-19, and studied the relation between level of these factors with severity and outcome of the disease.

**Results:**

Mean CRP level, creatinine, hemoglobin, and the leukocytes count in the critically ill patients were significantly higher than those of the other groups (non-critical patients); mean CRP = 54.81 mg/l, mean creatinine = 86.82 μmol/l, mean hemoglobin = 144.05 g/l, and mean leukocyte count = 7.41 × 10^9^. The lymphocyte count was higher in patients with mild/moderate disease (mean: 1.32 × 10^9^) and in the invasive ventilation group (mean value of 0.72 × 10^9^), but it was considerably lower than those of the other two groups. The results showed that the platelet count was higher in critically ill patients (mean value of 205.96 × 10^9^). However, the amount was lower in the invasive ventilation group compared with the other groups (mean level = 185.67 × 10^9^).

**Conclusion:**

With increasing disease severity, the leukocyte count and the level of CRP increase significantly and the lymphocyte count decreases. There seems to be a significant relation between platelet level, hemoglobin, and creatinine level with severity of the disease. However, more studies are required to confirm this.

## Background

In December 2019, an outbreak of pneumonia occurred in Wuhan, China [1]. Studies showed that the pneumonia is caused by a new coronavirus called severe acute respiratory syndrome coronavirus 2 (SARS-CoV-2) [2]. This new disease was named coronavirus disease 2019 (COVID-19) by the World Health Organization (WHO). It spread rapidly throughout China as well as other countries [3]. Coronaviruses infect a variety of animals (livestock, bat, and poultry) and humans. The disease can affect the respiratory, gastrointestinal, cardiovascular, and nervous systems [4–6]. Primary studies have shown that COVID-19 is more infective than SARS and MERS, but has a lower case fatality rate (CFR) [7]. A report from the Chinese Center for Disease Control and Prevention showed that the overall CFR for SARS-Cov-2 was 2.3% [8]. Higher CFR was detected in patients with comorbidities and who had the critical condition (the CFR was 49.0% among critical cases) [9–11].

Reports showed that patients who required ICU care were significantly older and were more likely to have underlying comorbidities such as hypertension, diabetes, cardiovascular and cerebrovascular diseases. There were also some laboratory differences between patients who were admitted in ICU and those admitted into regular units. For instance, patients who required ICU care had higher white blood cell and neutrophil counts, creatine kinase, and creatinine [11]. Moreover, according to the laboratory tests performed in the early stages of the disease, higher level of CRP has been reported in critically ill patients [12]. More severe lymphopenia and higher CRP levels were reported in a group of patients who did not survive [13]. Increased levels of hemoglobin and platelet have been seen in patients with severe condition who have received invasive mechanical ventilation. A study, unlike other studies, showed that thrombocytopenia that was seen in other virus infection, was not seen in COVID-19 patients on admission [14]. The CRP levels detected in severe patients have been higher than those of mild or moderate patients [12].

Quick recognition of potentially critical patients has an important role in the management of this disease [15]. In this article we aimed to show the differences of laboratory findings of COVID-19 patients with different severity and survivability who had been admitted in hospital. We can probably find a way to predict the prognoses of patients and help them as soon as possible.

## Methods

### Search strategy and selection criteria

we searched on an online database including PubMed, Scopus, Web of Science (WOS), Google Scholar from December to May 26, 2020, and followed this search strategy: ("COVID-19" OR "COVID-19 virus" OR "COVID19" OR "COVID19 virus" OR "SARS-COV-2" OR "2019-nCoV" OR "2019 novel coronavirus" OR "Wuhan coronavirus" OR "Novel coronavirus" OR "Coronavirus 2019" OR "coronavirus disease 2019") AND ("Creatinine" OR "Troponin" "Troponin I" OR "Cardiac troponin I" OR "cTnI" OR "Cardiac troponin T" OR "cTnT" OR "Creatine phosphokinase" OR "Creatine Kinase" "CpK" OR "Cpk-MB" OR "Creatine Kinase-MB" OR Crp OR "C-reactive protein" OR Leukopenia OR "Lymphopenia" OR "Hemoglobin" OR "Anemia" OR "Thrombocytopenia") AND (Intubation OR ICU OR Severe OR "CCU admission" OR Death OR Expire OR Hospitalize OR Hospitalization" OR Outcome).In this article, we selected the articles based on both inclusion and exclusion criteria. Inclusion criteria: 1) Study design included cross-sectional studies, Cohort studies, and case-control studies 2) patients have been identified as COVID-19 patients and were hospitalized 3) the presence of at least one of the laboratory findings reported in articles based on either disease severity (mild/moderate/severe/critical) or the ways of oxygen therapy. We defined disease severity based on Seventh Version of the Novel Coronavirus Pneumonia Diagnosis and Treatment Guidance from National Health Commission of China [16] 4) studies were reported in English languages. Ways of oxygen therapy were divided into three groups including: (1) invasive ventilation. (2) non-invasive ventilation. (3) no- oxygen therapy, which was defined as patients did not need anything to help them for breathing.

### Study selection

After EK removed duplicated study, EK, RS, FA screened the articles based on key word, abstract, and title. Then, we found the full text of the included articles and evaluated their eligibility for final inclusion.

### Data extraction and quality assessment

One author assessed the quality of included studies by "Appendix 2:MINORS criteria"[17]. The tool has 8 criteria for non-comparative and 4 additional criteria for comparative studies. The items are scored 0 (not reported), 1 (reported but inadequate), or 2 (reported and adequate). The global ideal score is 16 for non-comparative studies and 24 for comparative studies (Table 1). Data extraction was conducted by three investigators (EK, RS, FA). We used Microsoft excel databases to record our information including baseline details, laboratory examinations, disease severity (mild/moderate/severe/critical), outcome, type of oxygen therapy (invasive, non-invasive, no oxygen therapy).

**Table 1:** Minors criteria: A clearly stated aim /2: Inclusion of consecutive patients /3: Prospective collection of data / 4: Endpoints appropriate to the aim of the study /5: Unbiased assessment of the study endpoint /6: Follow-up period appropriate to the aim of the study /7: Loss to follow up less than 5%/ 8: Baseline equivalence of groups /9: Adequate statistical analyses

	1	2	3	4	5	6	7	8	9	Total score
Chen, Ruchong [38]	2	2	2	2	2	2	0	2	2	16
Guang Chen [22]	2	2	2	2	2	1	1	2	2	16
Xinyi Chen [34]	2	2	2	2	2	2	0	2	2	16
Davide Ferrari [45]	2	2	2	2	2	2	0	2	2	16
Jianhong Fu [32]	2	2	2	2	2	2	2	2	2	18
Wei Hou [43]	2	2	2	2	2	2	2	2	2	18
Chaolin Huang [31]	2	2	2	2	2	2	0	2	2	16
Hideaki Kato [27]	2	2	2	2	2	2	2	2	2	18
Huan Li [46]	2	2	2	2	2	1	2	2	2	17
Shaohua Li [23]	2	2	1	2	1	0	0	0	2	10
Claudio Liguori [47]	2	2	2	2	2	2	2	2	2	18
Hua Fan [13]	2	2	2	2	2	2	2	2	2	18
Jing Liu [37]	2	1	2	2	2	0	1	2	2	14
Kai Liu [30]	2	2	2	2	2	2	0	2	2	16
Tao Liu [41]	2	2	1	2	1	0	0	2	2	12
Zhihua Lv [24]	2	2	2	2	2	2	2	2	2	18
Giulia Rastrelli [39]	2	2	2	2	2	2	2	2	2	18
Weifeng Shang [48]	2	2	2	2	2	0	0	2	2	14
Ying Sun [20]	2	2	2	2	2	0	0	2	2	14
Chaochao Tan [12]	2	2	2	2	2	2	2	2	2	18
Suxin Wan [28]	2	2	2	2	2	2	2	2	2	18
Zhongliang Wang [29]	2	2	2	2	2	2	2	2	2	18
Ai-Ping Yang [33]	2	2	2	2	2	2	2	2	2	18
Jun Zhang [40]	2	2	2	2	2	1	0	2	2	15
Jin-jin Zhang [25]	2	2	2	2	2	2	2	2	2	18
Yafei Zhang [36]	2	2	2	2	2	2	2	2	2	18
Changcheng Zheng [44]	2	2	2	2	2	2	2	2	2	18
F. Zheng [26]	2	2	2	2	2	0	0	2	2	14
Yi Zheng [14]	2	2	2	2	2	2	2	2	2	18
Yaqing Zhou [21]	2	2	2	2	2	2	0	2	2	16
Yulong Zhou [42]	2	2	2	2	2	2	2	2	2	18
Jiaofeng Huang [49]	2	2	2	2	2	0	2	2	2	16
Shaobo Shi [51]	2	2	2	2	2	2	2	2	2	18
Mostafa Javanian [50]	2	2	2	2	2	2	2	2	2	18
Qingxian Cai [52]	2	2	2	2	2	2	2	2	2	18
Egon Burian [19]	2	2	2	2	2	1	0	2	2	15
Qingchun Yao [53]	2	2	2	2	2	2	2	2	2	18
Tobias H [35]	2	2	1	2	2	2	2	2	2	17

### Statistical analysis

The mean and standard deviations (SD) and its corresponding standard error (SE) were used to calculate the summary effects. The summary pooled mean with 95% CI was obtained using the random effects model. Cochran’s Q test was used to identify the heterogeneity of the results, and it was quantified using the I^2^ statistic. I^2^ statistic > 50% or Q statistics with p < 0·10 were considered as a significant between-study heterogeneity. We performed subgroup analysis by status, O2 therapy and study design. Moreover, between-study variance was assessed using the tau-squared (Tau^2^ or T^2^) statistic [18]. A jack-knife sensitivity analysis was conducted by removing the studies from meta-analyses one by one. Sensitivity analyses were also conducted to determine whether the results would change when one study was removed at a time. The results were fairly robust after removing the individual studies from the meta-analyses. Although the funnel plot was slightly asymmetric, after using the trim-and-fill method, visual inspection of the Begg funnel plot did not identify substantial asymmetry for the included studies. In addition, the Begg and Egger tests showed no evidence of publication bias (Begg test P = 0.32, Egger test P = 0.53). All statistical analyses were performed using STATA software (STATA; version 14). P-values below 0·05 were considered as statistically significant.

## Results

**Fig. 1:**
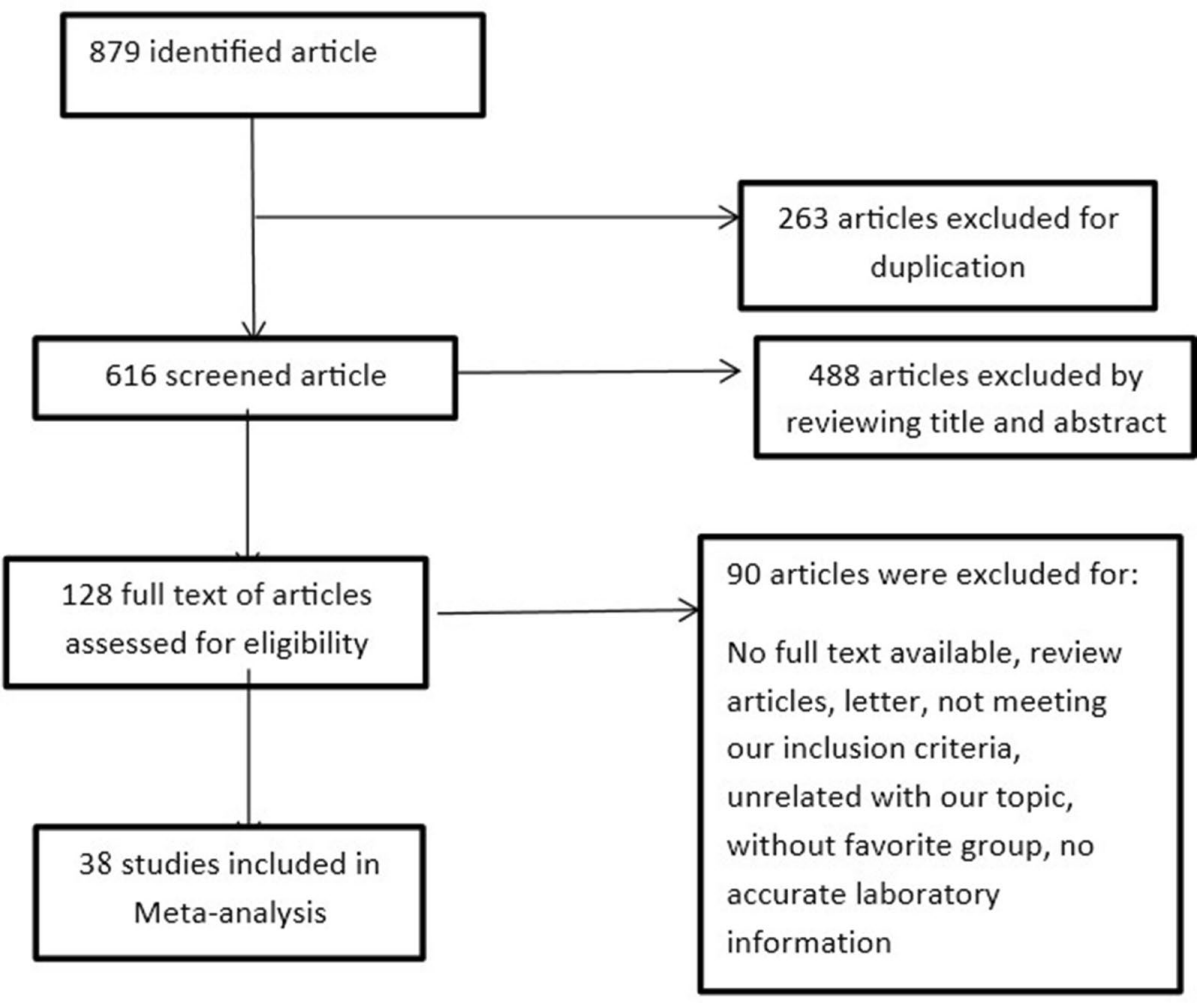
Forest plot showing the process of selected study

263 out of the 879 articles were excluded as the duplicated article. A total of 128 articles were obtained after screening based on title, keyword, and abstract. And 38 studies were included [12–14, 19–53] and 90 studies were excluded for this reasons: 24 articles were review, 11 articles were letter, 17 articles had the unrelated title, 18 articles were not based on our inclusion criteria, classification of patients were unusable for 4 articles, 12 articles did not have accurate laboratory information, we did not have access to 4 full texts (Fig. 1).

**Fig. 2:**
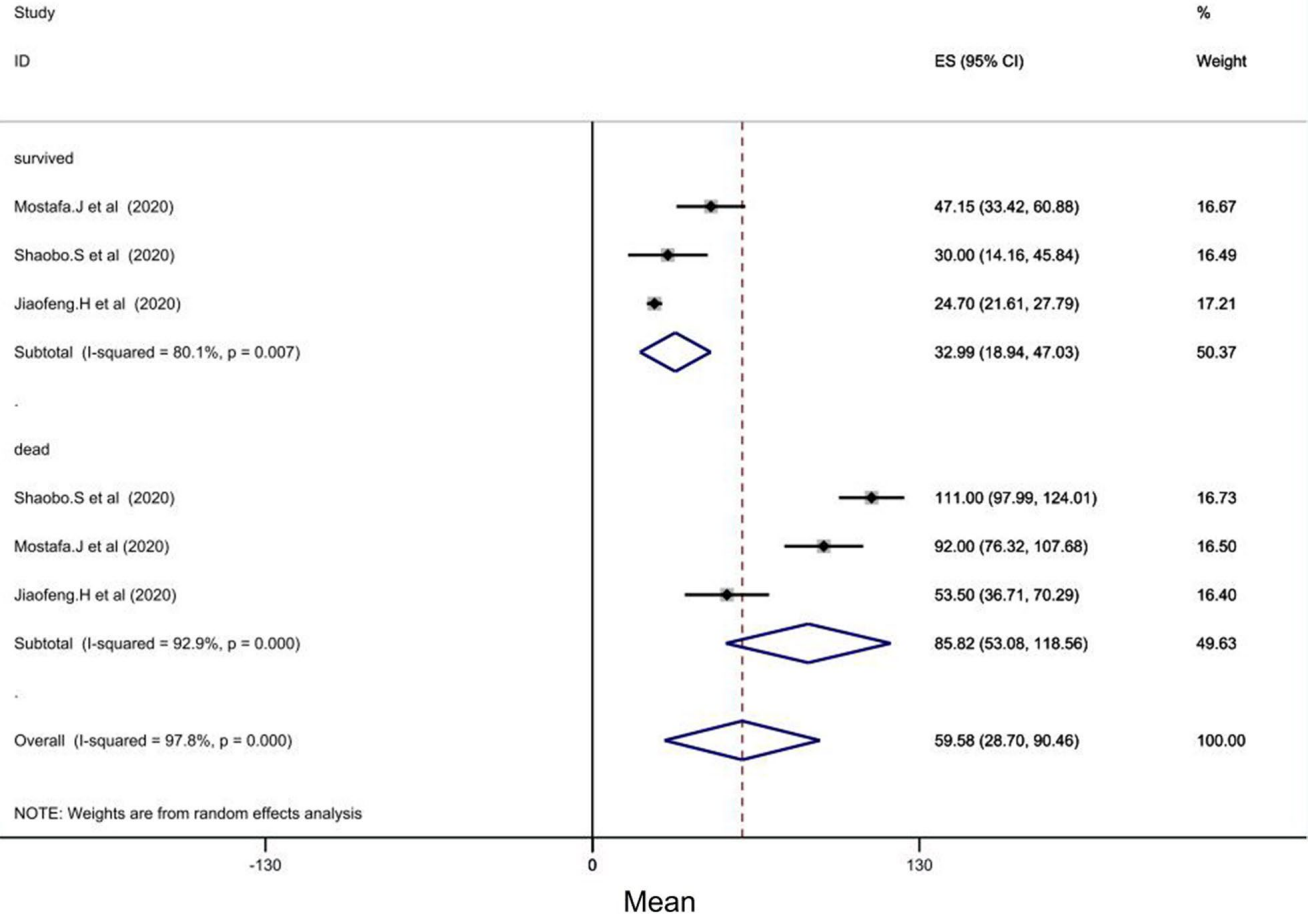
Forest plot showing pooled mean serum C-reactive protein (CRP) levels between patients with COVID-19 who survived (pooled average of 32.90 mg/l, 95%CI = [19.35, 46.45]) and those who died (pooled average of 85.82 mg/l, CI = [52.73, 118.91])

In 3 studies of 38 studies, CRP levels were assessed. The mean CRP between 97 COVID-19 patients that were dead, was 85.82 mg/l (CI = [53.08–118.56], P < 0.001), while the mean of CRP between 973 improved patients was 32.99 mg/l (CI = [18.94–47.03], P = 0.007) (Fig. 2).

**Fig. 3:**
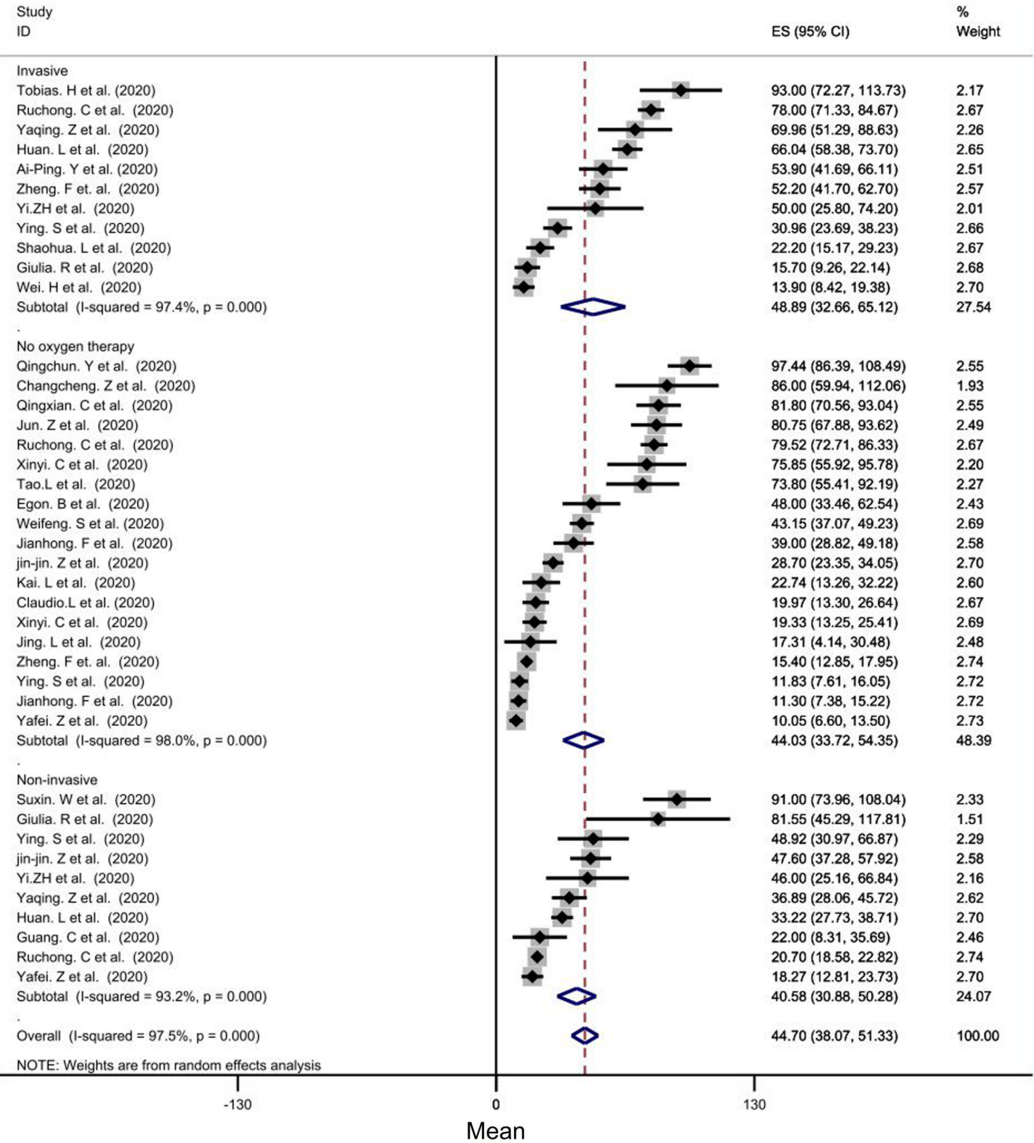
Forest plot showing pooled mean of CRP between groups according O2 therapy manner: (1) invasive ventilation group (pooled average of 48.83 mg/l, CI = [33.23, 64.42], p < 0.001), (2) non-invasive ventilation group (mean:42.40 mg/l), [ 27.99,56.81], p < 0.001), (3) no oxygen therapy group (pooled average of 44.59 mg/l), CI = [30.76, 58.43], p < 0.001)

Subgroup analysis, based on oxygen therapy, showed that the mean CRP of the invasive ventilation group (mean level = 48.89 mg/l, CI = [32.66–65.12], P < 0.001), in comparison with that of the non-invasive ventilation group (mean level = 40.58 mg/l) and the mean CRP of the no- oxygen therapy group (mean level = 44.03 mg/l), was significantly the highest. (Fig. 3).

**Fig. 4:**
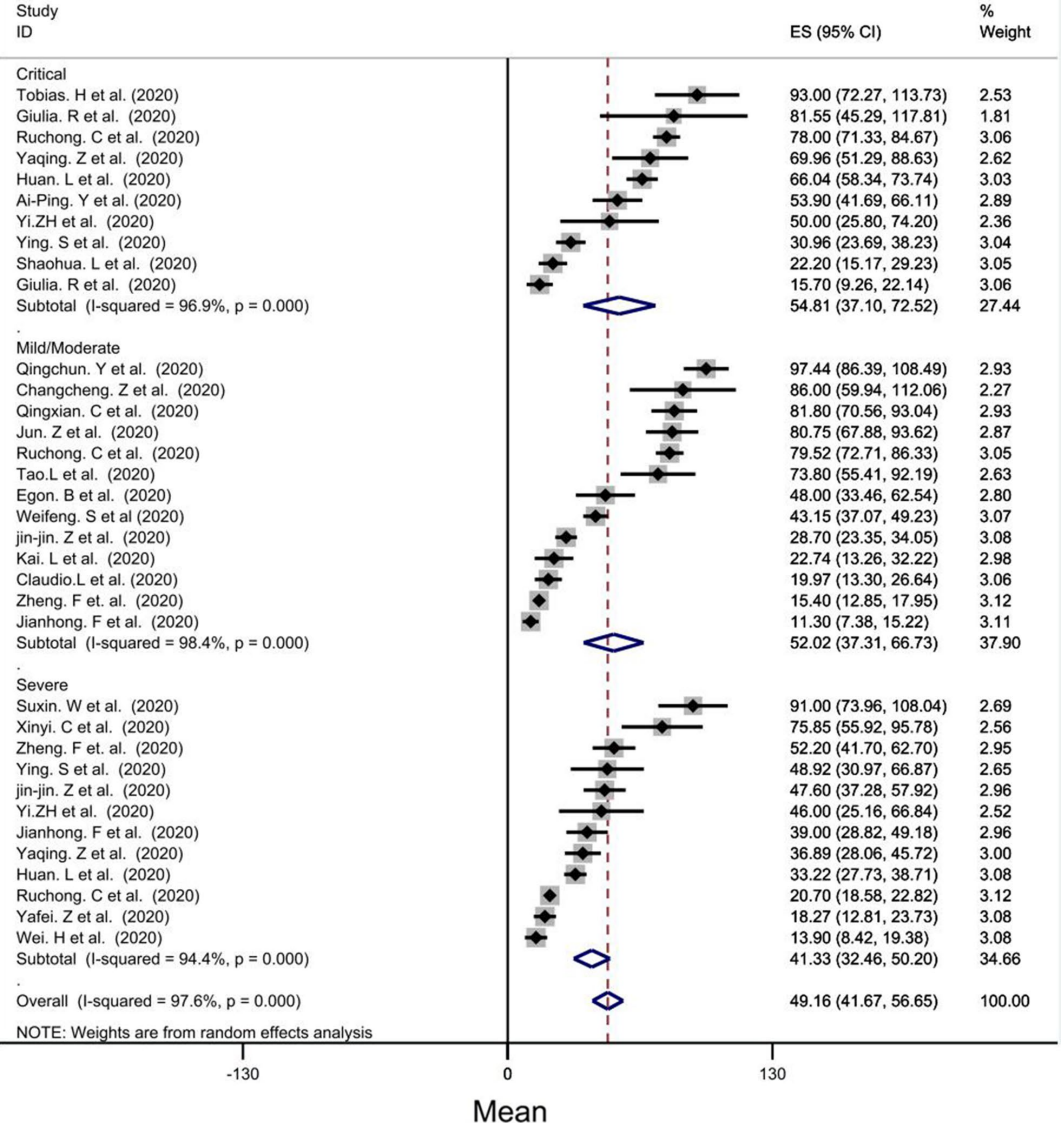
Forest plot showing pooled mean of CRP between groups according severity disease: (1) in critical group (pooled average of 54.65 mg/l, CI = [38.08–71.21], p < 0.001) was higher than other group (mild, moderate, severe), (2) in severe group (pooled average of 42.44 mg/l, CI = [29.92–54.96], p < 0.001) and (3) in mild group (pooled average of 14.74 mg/l)

Based on disease severity, the mean CRP of the critical group (mean: 54.81, CI = [37.10–72.52], P < 0.001) was the highest while the mild/moderte group had the second mean (mean level = 52.02 mg/l, CI = [37.31–66.73], P < 0.001) and the lowest mean was for severe group (mean level = 41.33 mg/l) (Fig. 4).

Subgroup analysis showed that the mean serum creatinine level in the critical group (86.82 μmol/l, CI [80.27–93.38], p < 0.001) was significantly higher than those in other groups. However, the mean creatinine of the severe group (mean level = 71.12 μmol/l, CI = [65.31–76.93], P < 0.001) was lower than that of the mild/moderate group. The mean for all of the patients was 78.19 (p value < 0.001) (Additional file 1: Fig. S1).

Subgroup analysis of leukocytes showed that the mean leukocytes in the critical group (mean level = 7.41 × 10^9^ per liter, CI = [6.75–8.07], P < 0.001) was higher than the mean leukocytes in other groups and had considerably a direct relationship with disease severity (Additional file 2: Fig. S2).

Based on analysis, the mean of lymphocytes in mild/moderate group (mean level = 1.32 × 10^9^ per liter, CI = [1.21–1.43], p < 0.001) was higher than those of the critical and severe groups and had a significant reverse relationship with disease severity (Additional file 3: Fig. S3).

The mean of platelet of all patients was 193.86 × 10^9^ per liter (CI = [184.37–203.36], P < 0.001). Subgroup analysis showed that the mean of platelet of the critical group (mean: 205.96 × 10^9^ per liter, CI = [185.86–226.05], p < 0.001) was higher than those of the other groups. However, it was lower in the severe group (mean: 177.38 × 10^9^ per liter, CI[163.48–191.28], p < 0.001) than those of the other groups (Additional file 4: Fig. S4).

Analysis of hemoglobin showed that the mean of hemoglobin in the critical group (mean: 144.05 g/l, CI = [131.57–156.53], p < 0.001) was higher than those of the other groups. Although this mean for the severe group was lower than that of the mild/moderate group, the mean of hemoglobin for all of the patients was significant (Additional file 5: Fig. S5).

The mean of creatinine for all of the patients was significant (mean level = 77.70 μmol/l, CI = [74.36–81.03], P < 0.001). Moreover, subgroup analysis demonstrated that the mean of creatinine in invasive ventilation group (mean level = 82.53 μmol/l, CI = [76.06–89.04], P < 0.001) was considerably higher than those of the other groups (Additional file 6: Fig. S6).

**Fig. 5:**
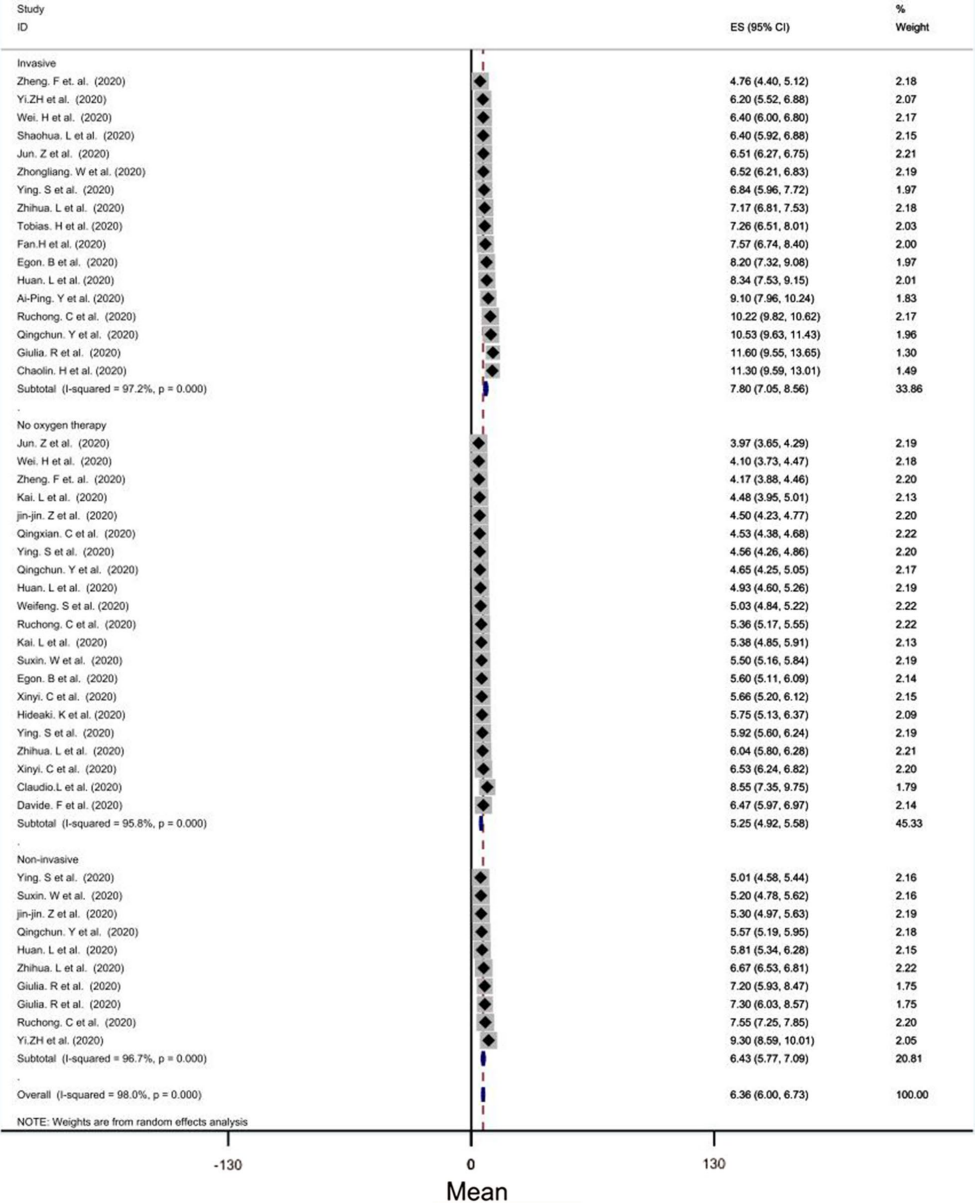
Forest plot showing pooled mean of leukocyte count between groups according O2 therapy manner: (1) invasive ventilation group (pooled average of 7.84 × 10^9^ per liter, CI = [6.94, 8.73], p < 0.001), (2) No oxygen therapy group (pooled average of 5.27 × 109 per liter), CI = [4.87,5.67] (3) Noninvasive O2 therapy group(pooled average of 6.45 × 109 per liter), CI = [5.60, 7.39]

The mean leukocytes of invasive ventilation group (mean level = 7.80 × 10^9^ per liter, CI = [7.05–8.56], P < 0.001) was significantly higher than those of the other groups. In addition, the mean leukocytes of no- oxygen therapy group (mean level = 5.25 × 10^9^ per liter) was lower than those of the other groups (Fig. 5).

Subgroup analysis showed that the mean lymphocytes of invasive ventilation -group (mean level = 0.72 × 10^9^ per liter, CI = [0.61–0.84], p < 0.001) was significantly lower than those of the other two groups. The mean of lymphocytes of patients who had a better condition and did not need oxygen therapy was higher than those of the other groups (Additional file 7: Fig. S7).

Our analysis showed the mean platelet for all of the patients (mean level = 193.16 × 10^9^ per liter, CI = [184.62–201.70], P < 0.001) was significant. Also the mean for each group was considerable. According to the analysis, the number of platelet of the invasive ventilation group was lower than those of the other groups (mean level = 185.67 × 10 ^9^ per liter) (Additional file 8: Fig. S8).

The mean hemoglobin of all patients was 131.44 g/l (CI [128.29, 134.58], p value < 0.001). Moreover, the mean hemoglobin was considerably higher in invasive -ventilation group (mean level = 139.66 g/l, CI = [131.35–147.98], P < 0.001) than those of the other groups (Additional file 8: Fig. S8).

## Discussion

The world is currently involved with a pandemic created by the new coronavirus, which has been detected in Wuhan, Hubei, since late December [54]. The disease has forced governments to take widespread actions to combat it, including quarantining thousands of millions of people all around the world. Due to the asymptomatic large number of patients, the outcomes of these actions were limited [45]. The clinical symptoms of these patients change rapidly, and the condition of severe patients can lead to hypoxia, concomitant organ failure, and death [55]. Although rapid identification of potentially critical individuals plays an important role in controlling the disease, there is still no definitive way to predict the severity and progression of the disease [15].

SARS-COV-2 infects humans through the airways by binding to the spike protein with human angiotensin-converting enzyme 2 which is present on the surface of renal cells and most alveolar (type 2) cells [56, 57]. Acute renal failure has been observed in patients whose clinical condition was poor, so this organ appears to be an important target for the virus [43, 58]. In the final stages of the disease, the clinical condition of patients deteriorates rapidly and abruptly. This exacerbation of clinical symptoms is accompanied by an increase in acute phase proteins (CRP, ESR), coagulation disorders and cell lysis, which can be caused by hyper inflammation [59–61]. According to laboratory results, leukopenia, lymphopenia, and thrombocytopenia were seen in some patients, but their prevalence was higher in SARS virus [28, 62].

Some studies have shown that an increase in CRP level may predict the severity of the disease, while this has not been the same result in some other studies. In an article by Yafei Zhang, results showed that there was no significant difference between the CRP levels after transferring the patients to ICU [36]. Although an increase in CRP level was seen in people with COVID-19 according to some study reports, based on statistical analysis, it was declared that this index may not be a good indicator of the expression of COVID-19 [45] However, a meta-analysis reported CRP level increased in severe form of COVID-19 [63]. Furthermore, In our study, based on our analysis, we found that non-survival individuals had higher CRP levels than survival people. We also found that as the severity of the disease increases, the level of CRP increases concurrently.

Two meta-analysis demonstrated that creatinine levels appear to be significantly associated with increased disease severity, and can be applied as a prognostic factor [63, 64]. The results of our study showed that the mean creatinine level increased with increasing disease severity, but this result was not true for all subgroups and it was found that in the severely ill group this average was lower, although this result may be due to the limitations of our study that will be mention later.

The results of some studies have shown higher leukocyte counts in critically ill patients [14, 37]. Whereas Yulong Zhou has found some contradictory results: the leukocyte counts were higher in individuals with severe disease, and there was another article with the result showing higher leukocyte counts in patients with lower inflammatory factors [42, 65]. In a meta-analysis performed by Takayuki Yamada, it was found that there was a direct and significant relationship between the number of leukocytes and the severity of the disease and leukocytosis (leukocyte count > 9.5 × 10^9^ per liter) was seen in people with severe disease significantly; [14] However, our analysis showed the opposite results.

The results of observations in several studies have emphasized that, to the extent that we can gauge lymphopenia (i.e., lymphocyte count < 1.5 × 10^9^ per liter) [20, 41], the more severe the disease, the lower the number of lymphocytes. In addition, a meta-analysis by Atieh.P et al. proved this fact [66]. Based on the findings observed in different groups of patients, we observed that the number of lymphocytes had a significant inverse relationship with the severity of the disease and lymphopenia was evident among all our subgroups in the study.

A study by Tao Liu reported that thrombocytopenia was not seen in non-severe patients, but there were some patients with thrombocytopenia (thrombocyte count < 1.5 × 10^9^) in the severe group, although this relationship was not significant [41]. Similar results were observed in other studies, where the mean platelet count was lower in people with more severe disease. However, the significant relationship was not proven [14]. Moreover, the meta-analysis published on 15 July implicated thrombocytosis as the most common lab finding in COVID-19 patients [67]. The results of our study showed that the number of platelets in critically ill patients was higher than the other groups, while this value was less for people with severe disease than the other groups. Analyses based on the type of oxygen therapy in the subgroups demonstrated that critically ill individuals had lower platelet counts. Although another meta-analysis showed that severe patients had lower platelets counts [68], there is a possibility about this controversial result of the study which is the definition of severity of disease, depending on what was originally defined in each included study. In our study, severity was defined based on Guidance from National Health Commission of China. Finally, it can be claimed that there seems to be a significant relationship between platelet count and disease severity. However, more studies are required in this area.

In a paper written by Tao Liu, Hemoglobin levels were reported lower in the severe patients than in non-severe patients [41], while there was an opposite result in another article [14, 48]. Based on our results, it seems that when the disease intensifies, the level of hemoglobin also increases, but we need more detailed studies for proving this fact.

## Limitations

Some of the articles had written just the mean of laboratory findings, but CI or SD (standard deviation) was not obvious. As a result, we could not use them. In a few studies, an accurate amount of laboratory findings were not obvious either. Therefore, we were obligated to exclude them. If these articles were usable, our results would probably become more accurate.

## Conclusion

In this meta-analysis, we assessed differences of some laboratory tests in COVID-19 disease with various severity. Based on our findings, we observed that the number of leukocytes increases more in critical patients. There is also a direct and strong relationship between CRP and the severity of the disease. However, there is a reverse significant relationship between counts of lymphocytes and the severity of the disease. It seemed there was a significant direct relationship between creatinine level and the severity of the disease. Moreover, because of some controversial results about hemoglobin and platelet, it seems that these factors need more surveys.

## Supplementary Information

**Additional file 1: Fig. S1.** Forest plot of included studies showing pooled analysis of serum creatinine level in COVID-19 patients with different outcomes (mild, moderate, severe, and critical).

**Additional file 2: Fig. S2.**Forest plot of included studies showing pooled analysis of leukocytes counts in COVID-19 patients with different outcomes (mild/ moderate, severe, and critical).

**Additional file 3: Fig. S3.**Forest plot of included studies showing pooled analysis of lymphocytes counts in COVID-19 patients with different outcomes (mild/ moderate, severe, and critical).

**Additional file 4: Fig. S4.**Forest plot of included studies showing pooled analysis of platelets counts in COVID-19 patients with different outcomes (mild/ moderate, severe, and critical).

**Additional file 5: Fig. S5.**Forest plot of included studies showing pooled analysis of hemoglobin level in COVID-19 patients with different outcomes (mild/moderate, severe, and critical).

**Additional file 6: Fig. S6.**Forest plot of included studies showing pooled analysis of serum creatinine level in COVID-19 patients with different oxygen therapy (invasive ventilation, non-invasive ventilation, no oxygen therapy).

**Additional file 7: Fig. S7.**Forest plot of included studies showing pooled analysis of lymphocytes counts in COVID-19 patients with different oxygen therapy (invasive ventilation, non-invasive ventilation, no oxygen therapy).

**Additional file 8: Fig. S8.**Forest plot of included studies showing pooled analysis of thrombocytes counts in COVID-19 patients with different oxygen therapy (invasive ventilation, non-invasive ventilation, no oxygen therapy).

**Additional file 9: Fig. S9.**Forest plot of included studies showing pooled analysis of hemoglobin level in COVID-19 patients with different oxygen therapy (invasive ventilation, non-invasive ventilation, no oxygen therapy).

## Data Availability

The data are available and can be provided upon request.

## Abbreviations

COVID-19: Coronavirus disease 2019
CRP: C-reactive protein
CFR: Case fatality rate
SD: Standard deviation
SE: Standard error
CI: Confidence interval
ICU: Intensive care unit
ESR: Erythrocyte sedimentation rate
