# Supplementary figures and images for: The laboratory findings and different COVID-19 severities: a systematic review and meta-analysis
Source: Ann Clin Microbiol Antimicrob. 2021 Mar 16;20:17. doi: 10.1186/s12941-021-00420-3 (PMC7962428; doi:10.1186/s12941-021-00420-3)

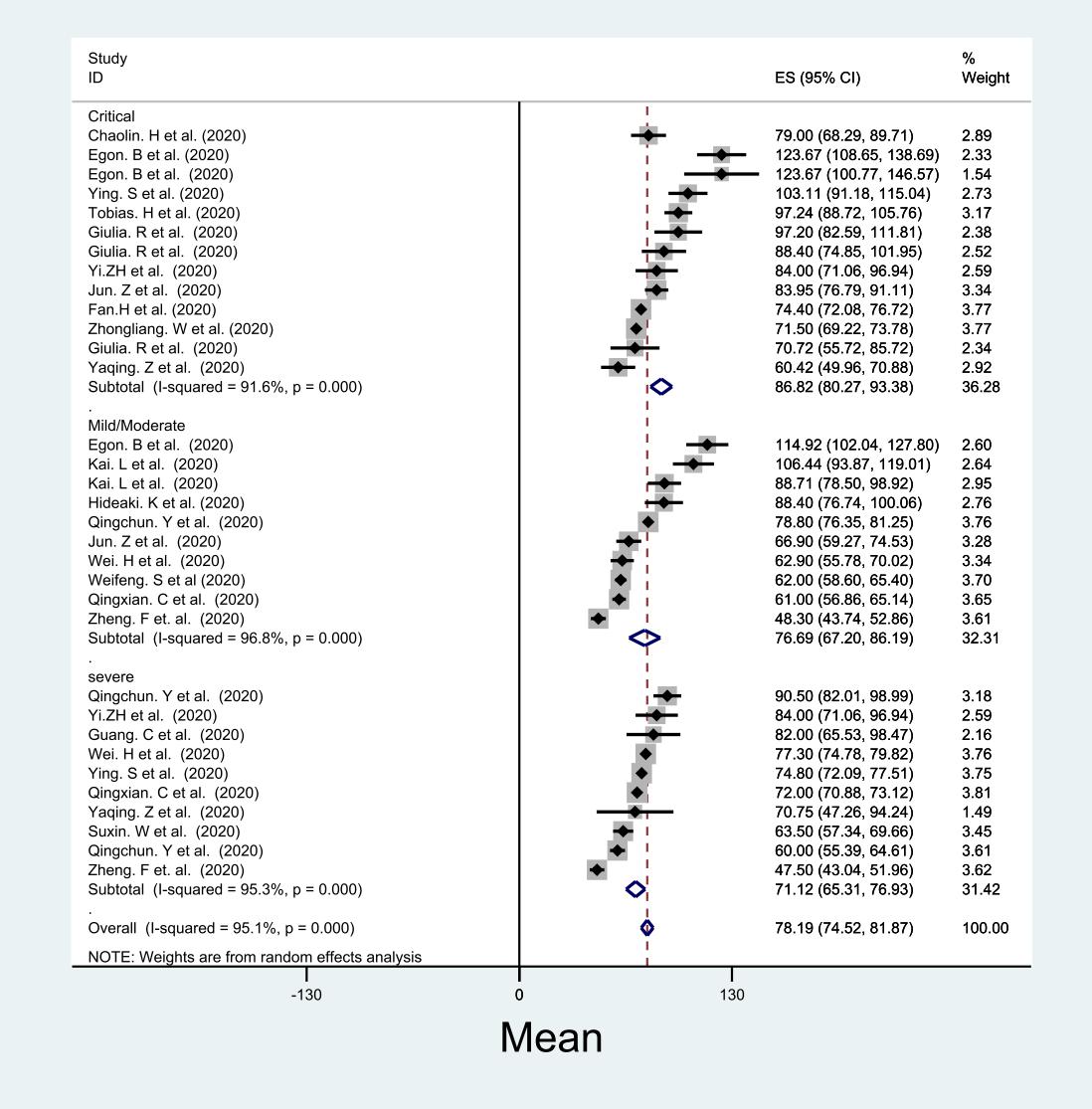

Supplement: Supplementary file 1 — Additional file 1: Fig. S1. Forest plot of included studies showing pooled analysis of serum creatinine level in COVID-19 patients with different outcomes (mild, moderate, severe, and critical). [file 12941_2021_420_MOESM1_ESM.jpg]

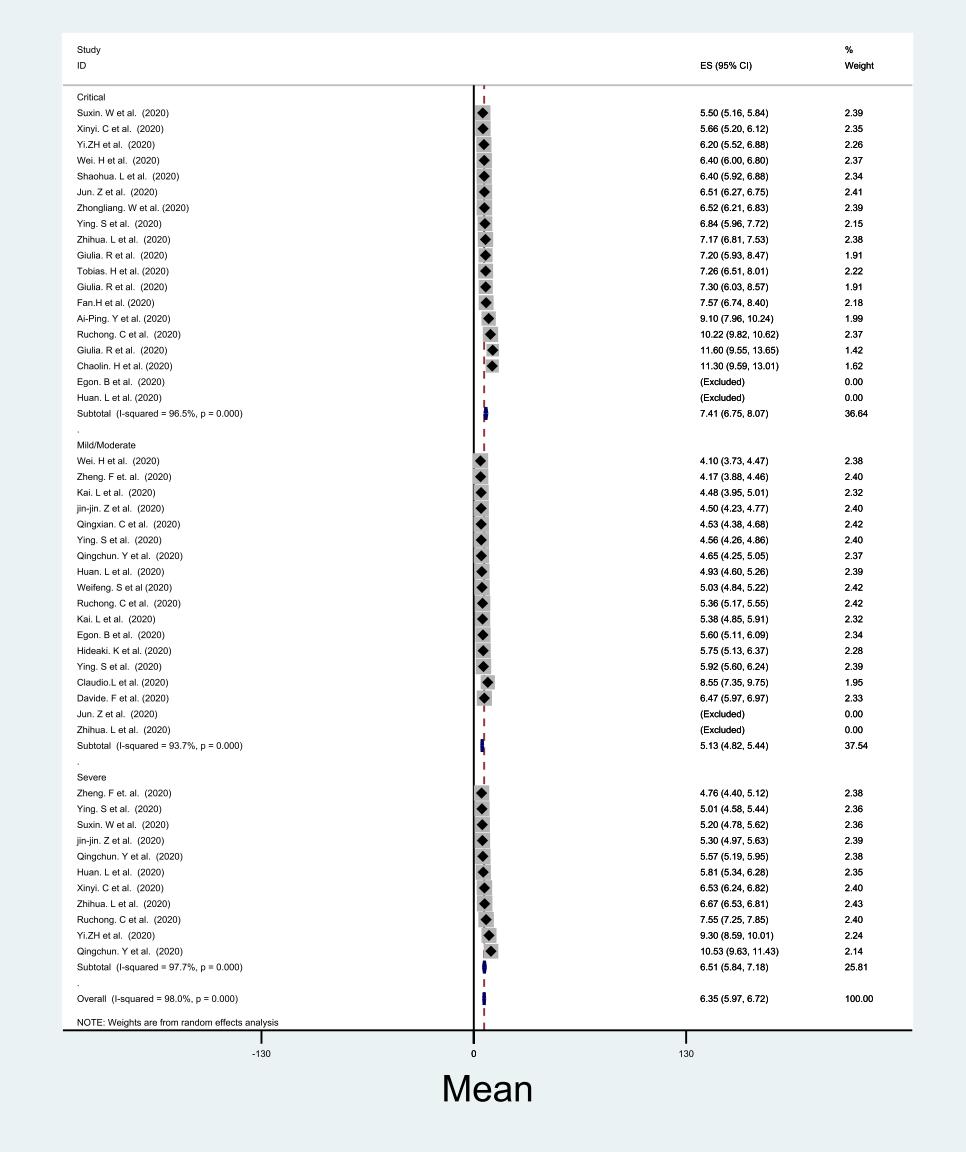

Supplement: Supplementary file 2 — Additional file 2: Fig. S2.Forest plot of included studies showing pooled analysis of leukocytes counts in COVID-19 patients with different outcomes (mild/ moderate, severe, and critical). [file 12941_2021_420_MOESM2_ESM.jpg]

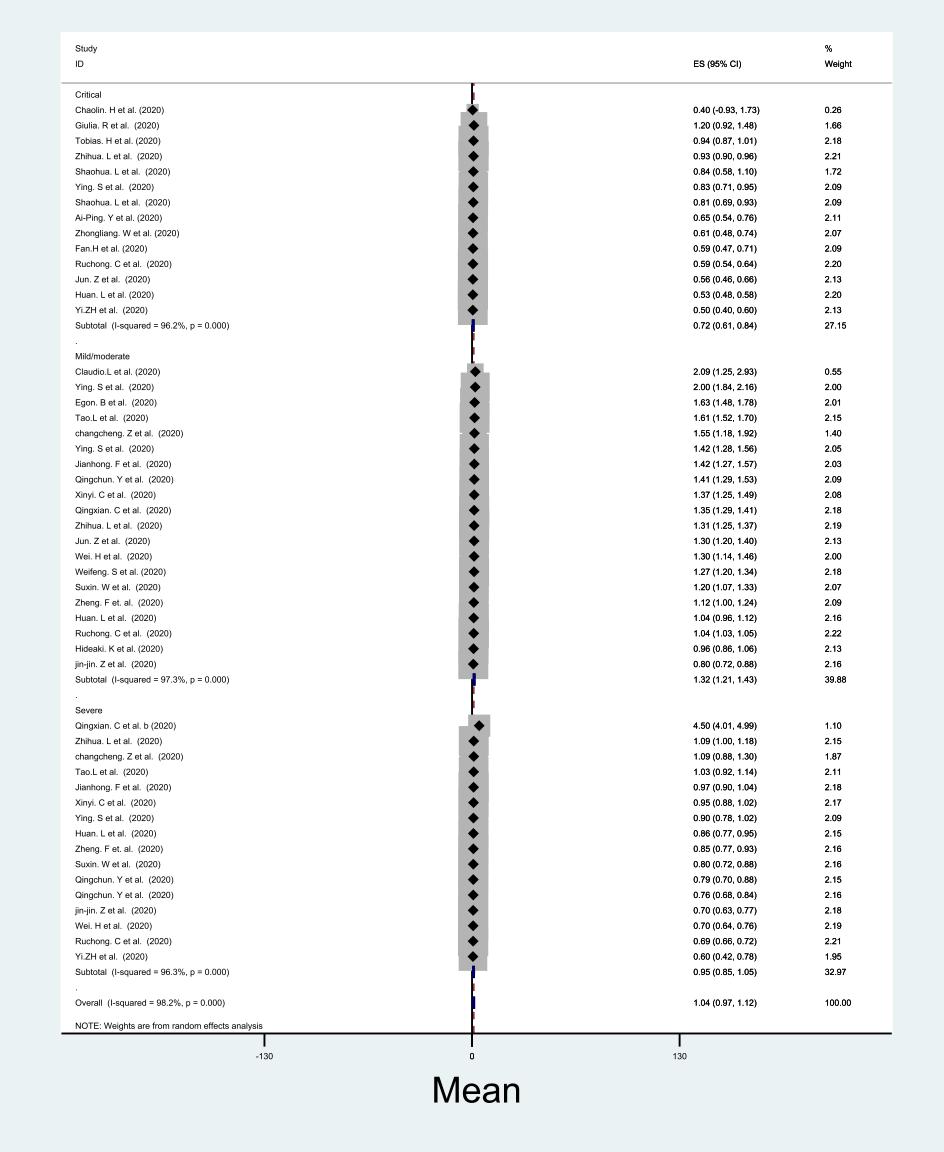

Supplement: Supplementary file 3 — Additional file 3: Fig. S3.Forest plot of included studies showing pooled analysis of lymphocytes counts in COVID-19 patients with different outcomes (mild/ moderate, severe, and critical). [file 12941_2021_420_MOESM3_ESM.jpg]

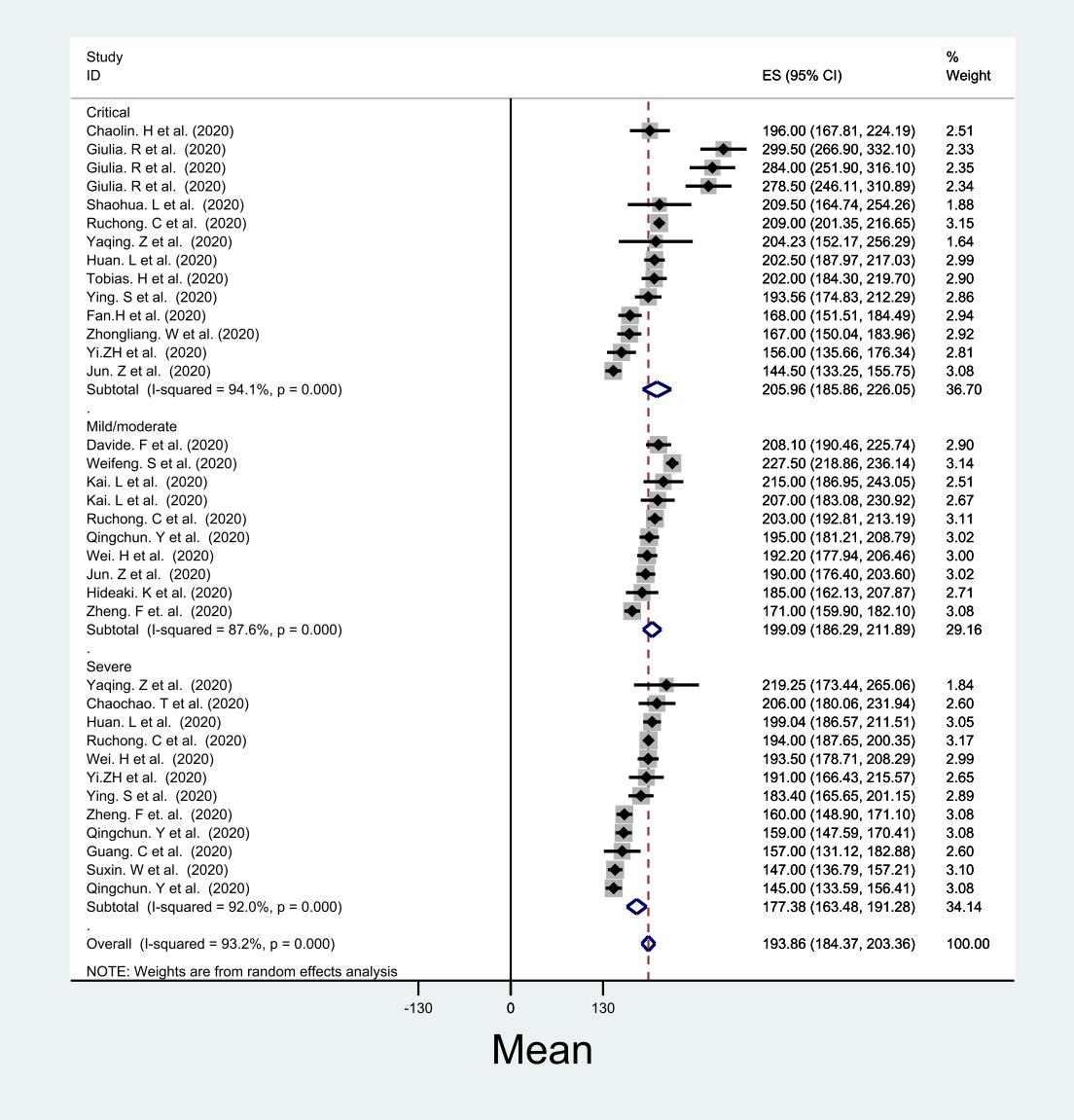

Supplement: Supplementary file 4 — Additional file 4: Fig. S4.Forest plot of included studies showing pooled analysis of platelets counts in COVID-19 patients with different outcomes (mild/ moderate, severe, and critical). [file 12941_2021_420_MOESM4_ESM.jpg]

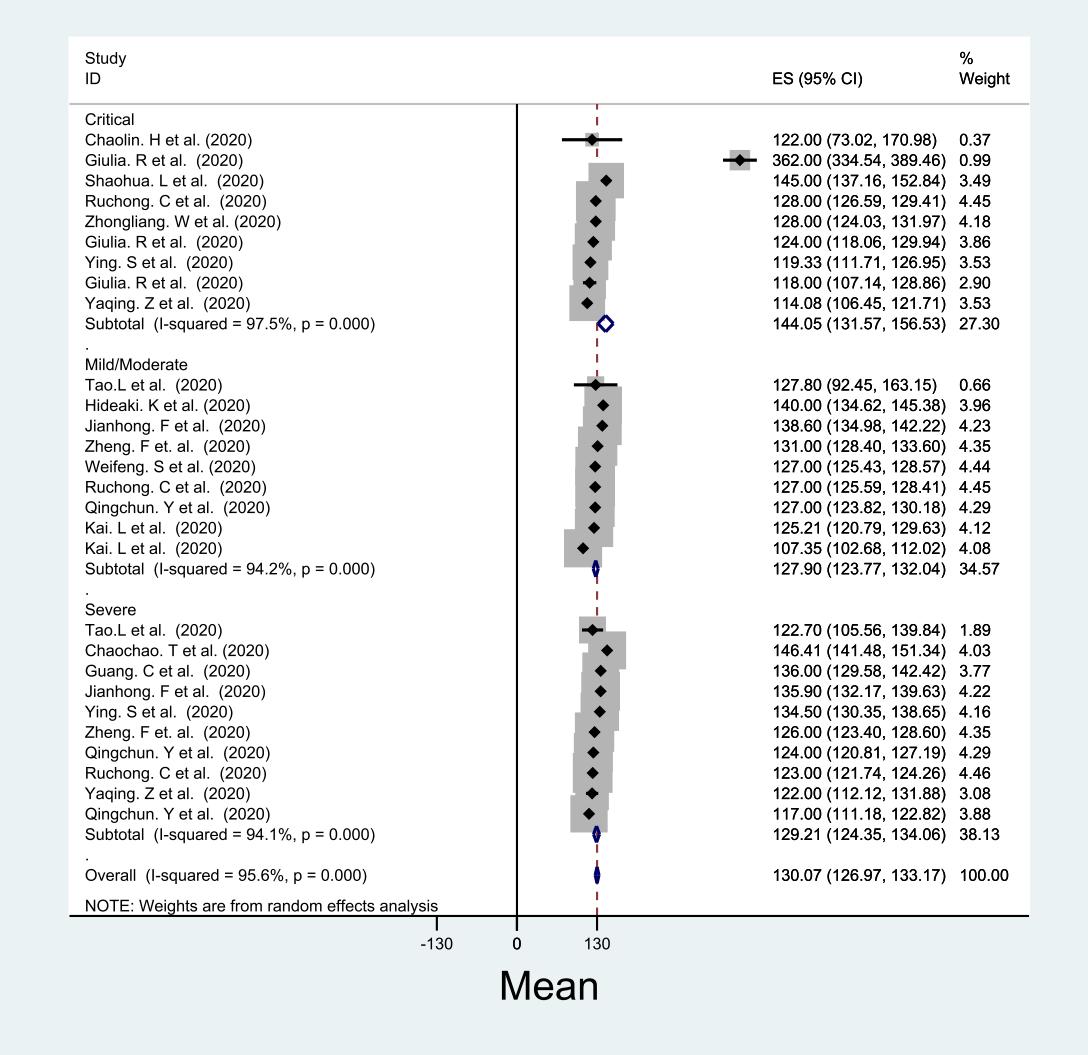

Supplement: Supplementary file 5 — Additional file 5: Fig. S5.Forest plot of included studies showing pooled analysis of hemoglobin level in COVID-19 patients with different outcomes (mild/moderate, severe, and critical). [file 12941_2021_420_MOESM5_ESM.jpg]

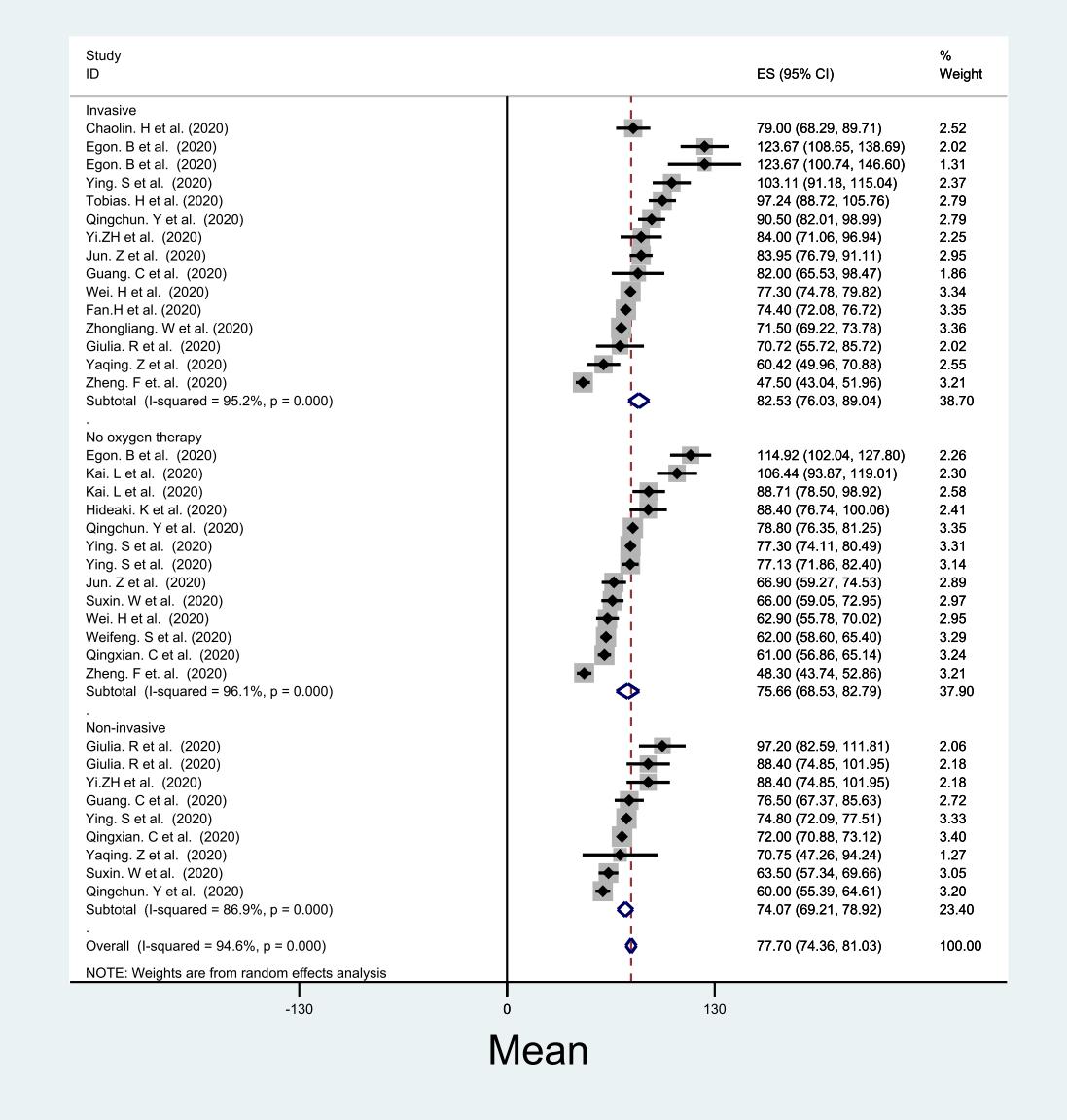

Supplement: Supplementary file 6 — Additional file 6: Fig. S6.Forest plot of included studies showing pooled analysis of serum creatinine level in COVID-19 patients with different oxygen therapy (invasive ventilation, non-invasive ventilation, no oxygen therapy). [file 12941_2021_420_MOESM6_ESM.jpg]

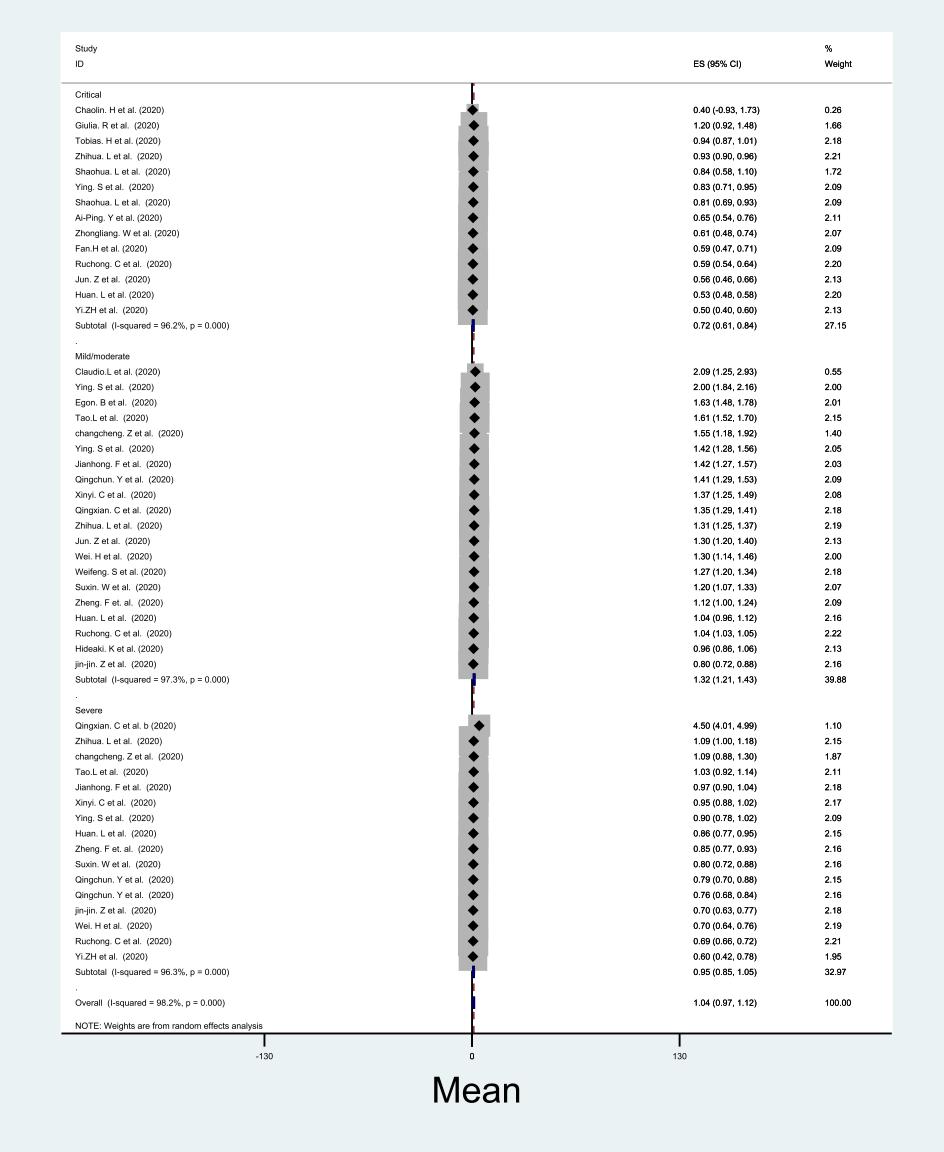

Supplement: Supplementary file 7 — Additional file 7: Fig. S7.Forest plot of included studies showing pooled analysis of lymphocytes counts in COVID-19 patients with different oxygen therapy (invasive ventilation, non-invasive ventilation, no oxygen therapy). [file 12941_2021_420_MOESM7_ESM.jpg]

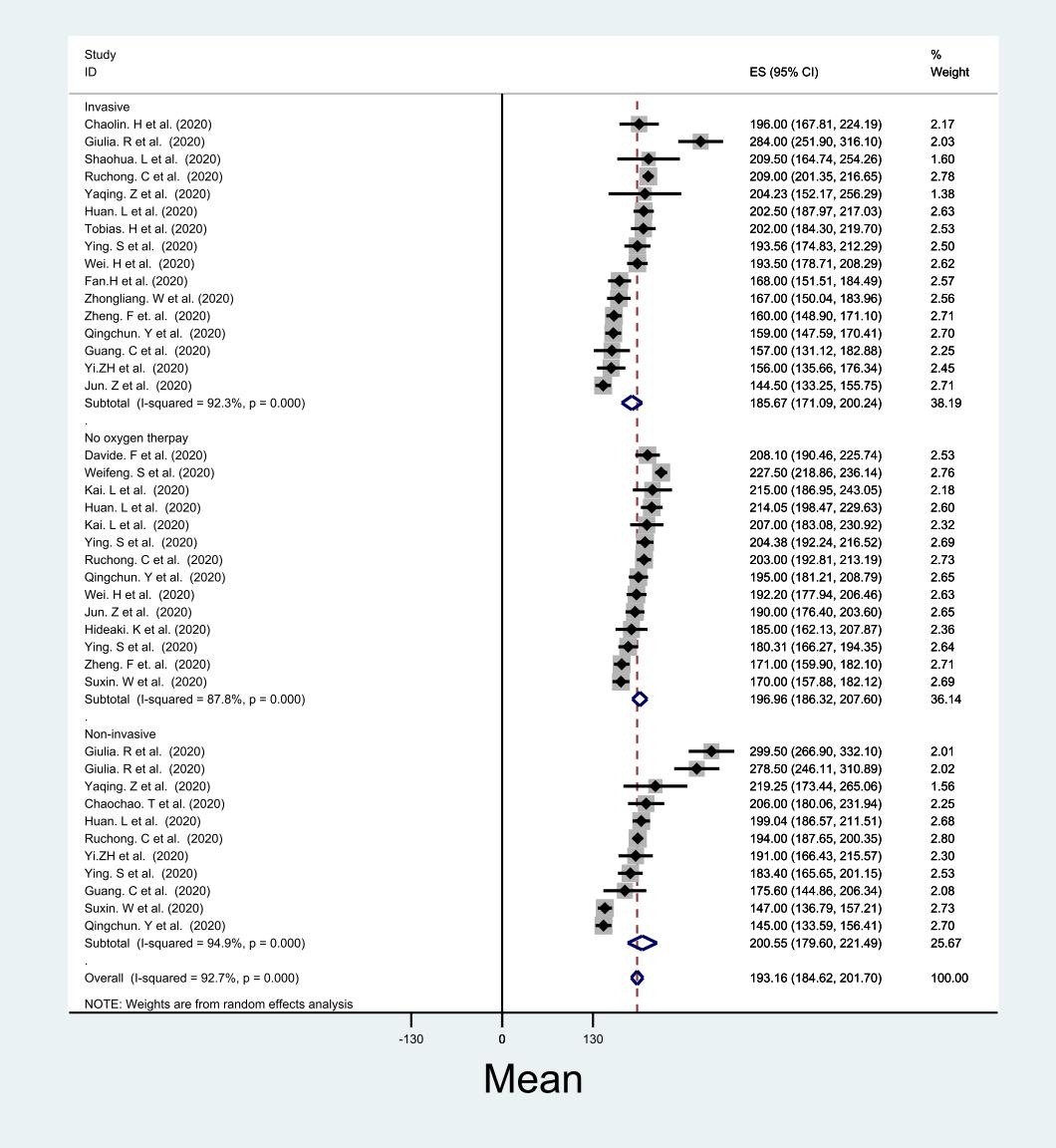

Supplement: Supplementary file 8 — Additional file 8: Fig. S8.Forest plot of included studies showing pooled analysis of thrombocytes counts in COVID-19 patients with different oxygen therapy (invasive ventilation, non-invasive ventilation, no oxygen therapy). [file 12941_2021_420_MOESM8_ESM.jpg]

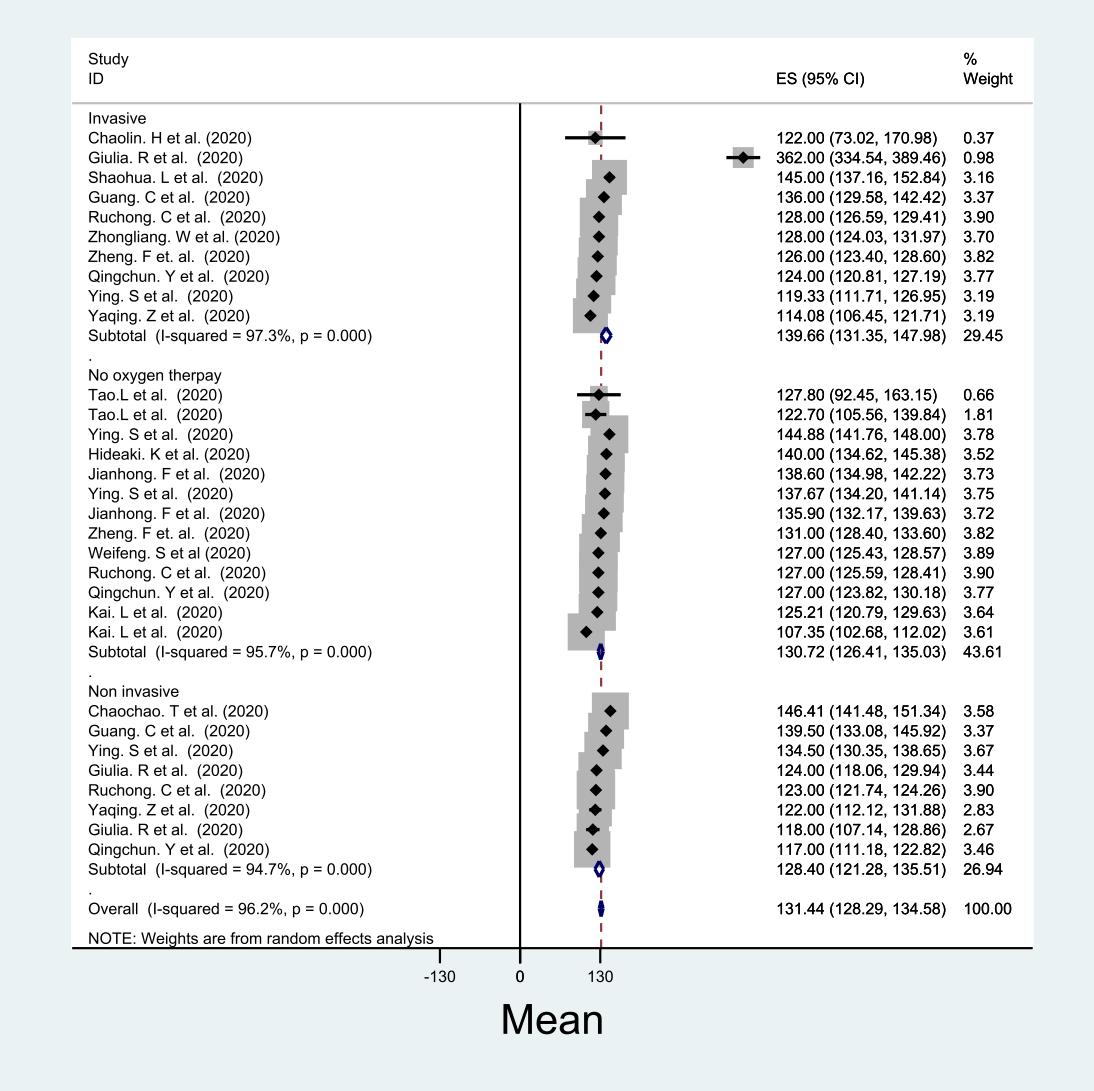

Supplement: Supplementary file 9 — Additional file 9: Fig. S9.Forest plot of included studies showing pooled analysis of hemoglobin level in COVID-19 patients with different oxygen therapy (invasive ventilation, non-invasive ventilation, no oxygen therapy). [file 12941_2021_420_MOESM9_ESM.jpg]
